# Evaluation of preoperative C-reactive protein aids in predicting poor survival in patients with curative colorectal cancer with poor lymph node assessment

**DOI:** 10.3892/ol.2013.1308

**Published:** 2013-04-16

**Authors:** YUJI TOIYAMA, HIROYUKI FUJIKAWA, YUKI KOIKE, SUSUMU SAIGUSA, YASUHIRO INOUE, KOJI TANAKA, YASUHIKO MOHRI, CHIKAO MIKI, MASATO KUSUNOKI

**Affiliations:** Department of Gastrointestinal and Pediatric Surgery, Mie University Graduate School of Medicine, Tsu, Mie 514-8507, Japan

**Keywords:** colorectal cancer, C-reactive protein, lymph node number, lymph node ratio, stage migration

## Abstract

Lymph node status is the most significant prognostic factor of colorectal cancer. However, there is a risk of disease understaging if the extent of lymph node assessment is sub-optimal. Preoperative C-reactive protein (CRP) is known to be a useful tool in predicting postoperative outcomes in patients with colorectal cancer. We retrospectively evaluated whether CRP adds to prognosis information in stage I–III colorectal cancer patients with poor lymph node assessment. In stages I–III, multivariate analysis revealed that CRP-positive status and advanced T-stage were factors that independently affected survival. In stage III, univariate analysis revealed that lymph node number retrieval and lymph node ratio were factors that affected survival. However, CRP positivity was the only independent factor for survival. CRP positivity did not predict poor prognosis in stage II or III patients with adequate lymph node retrieval. By contrast, the prognosis of CRP-positive patients was poorer than that of CRP-negative patients in stage II and III, with inadequate lymph node retrieval. CRP is an independent prognostic marker in patients with stage I–III, II or III colorectal cancer. The evaluation of CRP may provide useful information on prognosis in curative patients with an inadequate examination of lymph nodes.

## Introduction

Accurate assessment of the presence of lymph node metastasis is critical in predicting the clinical outcome of patients who have undergone radical surgery for colorectal cancer. The status of the lymph nodes also largely determines whether adjuvant chemotherapy should be administered. Such adjuvant chemotherapy has been shown unequivocally to provide disease-free, as well as overall survival benefits, in patients with node-positive disease (1). The current literature indicates that the accuracy of staging and overall survival in colon cancer increases proportionally with the number of lymph nodes examined (2–4). However, recent publications have suggested substantial variation in nodal staging, attributable to surgical, pathological and patient factors (3–5). These clinicopathological factors may result in inaccurate staging and subsequent inappropriate therapy. Previous studies focusing on stage II disease showed that for patients with negative nodes, the survival rate is lower when relatively few lymph nodes are recovered and examined, indicating that these patients may be understaged (4,6,7). Therefore, the current American Joint Committee on Cancer (AJCC) guidelines suggest that a minimum of 12 nodes be present in the surgical specimen (8).

By contrast, there are conflicting data regarding lymph node retrieval in stage III disease. Prandi *et al*(9) reported that overall and relapse-free survival in stage III patients was not correlated to the number of lymph nodes recovered. A similar conclusion was also reported by Wong *et al*(10) who stated that the number of lymph nodes following colectomy is not associated with patient survival at a hospital level and that a higher number of retrieved nodes might not be of public health value.

The lymph node ratio (LNR), which is the ratio of metastatic lymph nodes to examined lymph nodes, has been proposed as a potentially more accurate predictor of overall survival (OS) and disease-free survival (DFS) in colorectal as well as gastric cancer. Noura *et al* reviewed the literature demonstrating the clinicopathological significance of LNR in colorectal cancer patients, and revealed that several reports have indicated the advantage of considering the LNR compared with the number of lymph nodes retrieved and/or lymph node status. The cut-off points for LNRs were proposed in numerous studies; however, a consensus has not yet been reached with regard to optimal thresholds for LNRs (11).

C-reactive protein (CRP) is an acute phase reactant that acts as a surveillance molecule for the activation of the adaptive immune system. It is synthesized in hepatocytes and is upregulated by cytokines such as interleukin (IL)-6 and tumor necrosis factor-α (12). Several studies have demonstrated that elevated CRP levels are associated with an increased risk of early recurrence and poor outcome following colorectal cancer resection (13–15). Previously, we also reported that CRP levels reflect IL-6 production in colorectal cancer tissues and predict poor prognosis in colorectal cancer patients, particularly in stage I or II patients who are not usually candidates for postoperative adjuvant chemotherapy (16,17).

Therefore, in this study, we examined whether elevated CRP levels could be used to identify a subset of patients with very poor prognosis in stage II or III colorectal cancer. We also assessed whether elevated CRP levels could provide additional information concerning stage migration, which is necessary when inadequate retrieval of lymph nodes in stage II or III colorectal cancer occurs.

## Materials and methods

### Patients

Overall, 193 patients with stage I–III colorectal cancer who received potentially curative surgery at our institution between January 1995 and January 2005 were enrolled in this retrospective study. Curative resection was defined as the absence of any gross residual tumor from the surgical bed and a surgical resection margin that was pathologically negative for tumor invasion. Data were retrieved from operative and pathological reports. Follow-up data were obtained from the outpatient clinical database. Blood collection and subsequent analyses were approved by the Institutional Review Boards of Mie University Graduate School of Medicine (protocol number: 2216).

### Tumor characteristics

The study group comprised of 116 males and 77 females aged 29–91 years (median, 66 years; interquartile range, 58–73 years). Staging was principally based on the UICC/TNM classification of colorectal cancer. Overall, 42 patients had stage I disease, 74 patients had stage II and 77 had stage III disease. Experienced pathologists from our institution participated in this study and reassessed the quality of the original diagnosis. Of the 193 registered patients, 104 had tumors located in the colon and 89 had tumors in the rectum. The pathological tumor diameter indicated the maximum micro-scopic length of the tumor, irrespective of the depth. Differentiated tumors were histologically observed in 178 patients and undifferentiated tumors in 15 patients. Lymphatic invasion was observed in 174 patients and vascular invasion in 97 patients. After 1997, 68 patients who gave informed consent received adjuvant chemotherapies. Starting 4 weeks after curative surgery, pyrimidine fluoride-based regimens were used for 6 months to 1 year in patients classified as mainly stage III [stage II, 26/74 (35.1%); stage III, 42/77 (54.5%)].

### Methods

Patients were followed up, using our standard protocol, every 12–16 weeks for at least 5 years. This protocol included tumor-marker studies, computed tomography, colorectal fiber examinations, ultrasonography and chest radiography. Bone scans were performed when bone metastasis was indicated. The mean follow-up time was 59.5 months (95% CI for the mean was 54.5–64.5 months). The clinicopathological parameters studied for prognostic value were tumor size, T classification, vessel involvement, lymphatic invasion, lymph node metastases and serum carcinoembryogenic antigen (CEA) concentration.

Blood samples were collected for routine laboratory measurements of CRP prior to surgery. This is standard practice for all cancer patients in our institution. The coefficient of variation for these methods, over the range of measurement, was <5%, as established by routine quality control procedures. Patients who underwent non-elective surgery or preoperative radiotherapy, who died within 30 days of surgery, or who showed clinical evidence of infection or other inflammatory conditions, were excluded from the study.

**Figure 1:**
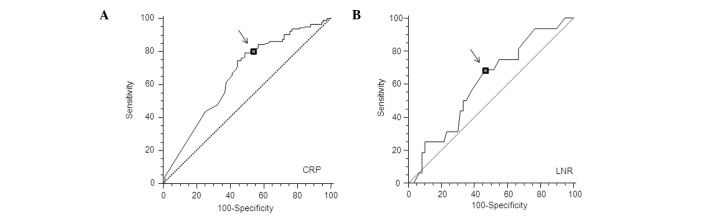
(A) ROC curves for peak serum CRP in patients with stage I–III colorectal cancer (n=191). (B) ROC curves for peak LNR in stage III colorectal cancer (n=77). The arrows indicate the location on the ROC curves for the diagnostic cut-off point, thus minimizing misclassification of surviving and deceased patients (CRP: sensitivity 79.5%, specificity 51.1%, cut-off 0.5 mg/dl; LNR: sensitivity 68.7%, specificity 53.3%, cut-off 0.15). CRP, C-reactive protein; LNR, lymph node ratio.

In the present study, elevated serum CRP levels were defined according to the best predictive values calculated by receiver operating characteristic (ROC) analyses, which found the best pair of values for highest sensitivity and specificity based on the peak and cut-off points (Fig. 1A). Based on this analysis, the cut-off value for CRP was calculated to be 0.5 mg/dl.

### Statistical methods

The data were presented as the means ± standard deviation (SD). Comparisons were made using the non-parametric Mann-Whitney U test for continuous variables and the χ^2^ test for categorical data. Correlations were analyzed by Spearman’s coefficient analysis. ROC analyses were performed to calculate the cut-off values according to the most accurate value obtained using Medcalc 7.2 for Windows (Mariakerke, Belgium). The survival probabilities were calculated using the product limit method of the Kaplan-Meier method of analysis, considering treatment-related mortality and mortality caused by colorectal cancer. The differences between the two groups were determined using the log-rank test. The effect of each significant predictor identified by the log-rank tests was assessed by multivariate analysis using Cox’s proportional hazard model. Statistical analyses were carried out using StatView 5.0 (SAS Institute Inc., Cary, NC, USA) for Windows. Two-sided p-values of <0.05 were considered to indicate statistical significance.

## Results

### Correlation between CRP and clinicopathological characteristics in patients undergoing potentially curative resection for colorectal cancer

During the observation period, 31 patients succumbed to colorectal cancer. Overall, 45 patients had elevated CRP levels (>0.5 mg/dl). The mean number of lymph nodes examined was 12.6±0.75 in stage I–III colorectal cancer. The mean number of positive lymph nodes and LNR in stage III colorectal cancer were 2.6±0.2 and 0.23±0.02, respectively. Table I shows the correlation between clinicopathological characteristics and CRP status in patients with stage I–III colorectal cancer. Gender, vascular or lymphatic invasion, lymph node metastasis, pathological differentiation and TNM classification were not significantly associated with CRP concentration. However, age, serosal invasion and CEA levels were significantly associated with CRP concentration.

### Univariate and multivariate analyses in relation to mortality in patients with stage I–III colorectal cancer

**Figure 2:**
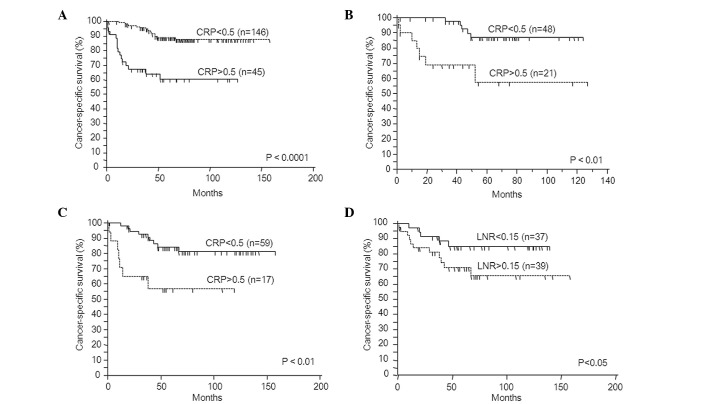
Kaplan-Meier analysis of cancer-specific survival rates of patients with stage I–III (A), II (B) and III (C) patients, subdivided by CRP status. (D) Kaplan-Meier analysis of cancer-specific survival rates of stage III patients subdivided by LNR cut-off value. CRP, C-reactive protein; LNR, lymph node ratio.

The results of the univariate and multivariate analyses of postoperative mortality are shown in Table II. Based on Cox’s univariate proportional hazard model, serosal invasion (p= 0.03), undifferentiated tumors (poorly differentiated and mucinous adenocarcinoma; p= 0.013), elevated serum CEA levels (p= 0.0008) and CRP positivity (p<0.0001) were significant prognostic factors for poor survival in patients with stage I–III colorectal cancer. Multivariate analysis revealed that undifferentiated tumors (p=0.002) and CRP detection (p<0.0001) were the only independent risk factors for predicting poor prognosis. Fig. 2A shows cancer-specific survival according to the CRP status. CRP-positive patients had a significantly worse prognosis than patients whose levels were below the cut-off value (log-rank test, p<0.0001).

### Correlation between survival and clinicopathological findings, including lymph node number examined and CRP status, in stage II colorectal cancer

The cancer-specific survival rate was not significantly higher for patients with 12 or more lymph nodes examined compared with those with <12 lymph nodes examined (log-rank test, p= 0.09; ≥12, 92.0%; <12, 77.2%). The results of univariate and multivariate analysis of postoperative mortality in stage II are shown in Table III. Based on Cox’s univariate proportional hazard model, serosal invasion (p= 0.024) and CRP presence (p= 0.005) were significant prognostic factors for poor overall survival. Multivariate analysis revealed that CRP (p= 0.02) was the only independent risk factor for predicting poor prognosis. Fig. 2B shows the cancer-specific survival according to the CRP status. CRP-positive patients had a significantly poorer prognosis than patients whose levels were below the cut-off value (log-rank test, p<0.01; cancer-specific survival rate: CRP-positive, 89.5%; CRP-negative, 66.6%).

### Correlations between survival and clinicopathological findings, including number of lymph nodes examined, pN stage, LNR and CRP status in stage III colorectal cancer

In the patients with stage III colorectal cancer, we determined an optimal cut-off value for the LNR as it was not previously defined. Fig. 1B shows that the optimal LNR cut-off value was 0.15 by ROC analysis. By contrast, optimal cut-off values for the number of lymph nodes examined and pathological N stage were 12 and 4, respectively. These values were recommended or stated in the AJCC guidelines (8) and TNM classification (27).

The cut-off value for the total number of lymph nodes retrieved and the pN did not alter the cancer-specific survival rate significantly (log-rank test: total number of lymph nodes retrieved, p=0.1008; pN, p=0.784). By contrast, CRP-positive patients had a significantly poorer prognosis than patients whose levels were below the cut-off value (log-rank test, p<0.01; Fig. 2C). Using a cut-off value for LNR of 0.15 also maintained significance for cancer-specific survival (log-rank test, p<0.05; Fig. 2D). The results of the univariate and multivariate analyses of postoperative mortality in stage II are shown in Table IV. Based on Cox’s univariate proportional hazard model, LNR >0.15 (p=0.033), CEA >6 (p=0.01) and CRP positivity (p=0.008) were significant prognostic factors for poor cancer-specific survival. Multivariate analysis revealed that CRP levels (p=0.028) were the only independent risk factor for predicting poor prognosis.

### Evaluation of preoperative CRP predicts understaging in patients is correlated with poor prognosis and occurs due to sub-optimal lymph node assessment in stage II or III colorectal cancer

**Figure 3:**
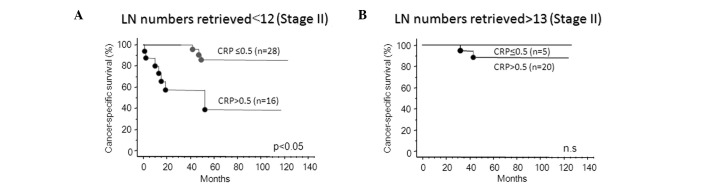
Kaplan-Meier analysis of cancer-specific survival rates of stage II patients subdivided by CRP status and by lymph node number. (A) Lymph node number <12. (B) Lymph node number >13. CRP, C-reactive protein; LN, lymph node.

**Figure 4:**
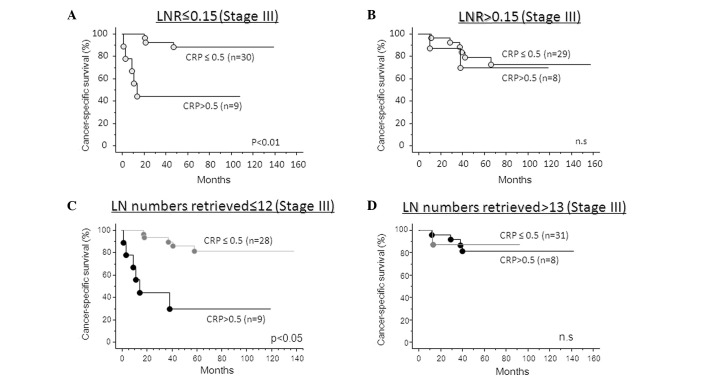
Kaplan-Meier analysis of cancer-specific survival rates in stage III patients subdivided by CRP status combined with LNR or number of lymph nodes retrieved. (A) LNR <0.15; (B) LNR >0.15; (C) Lymph node number <12; (D) Lymph node number >13. CRP, C-reactive protein; LN, lymph node; LNR, lymph node ratio.

Fig. 3A shows that, in stage II colorectal cancer, an adequate number of lymph nodes examined resulted in a relatively good prognosis. Therefore, recurrent patients were not predicted using CRP status. By contrast, CRP positivity significantly predicts the risk of recurrence if an inadequate number of lymph node are examined in patients that demonstrate poor prognosis (Fig. 3B). In addition, the CRP-positive group had significantly poorer cancer-specific survival compared with the CRP-negative group in stage III colorectal cancer patients with an inadequate number of examined lymph nodes (<12) or LNR <0.15 (log-rank test; lymph node number examined, p<0.05; LNR, p<0.01; Fig. 4A and C). By contrast, the incorporation of CRP status was not required when adequate lymph node numbers were examined (>13) or when LNR was >0.15 (Fig. 4B and D).

## Discussion

The TNM staging system provides the most reliable information on prognosis and aids in the discrimination of patients with early stage versus advanced stage disease. However, it is less accurate in predicting the prognosis of patients with an intermediate extent of tumor invasion. CEA is a complex glycoprotein that is upregulated in approximately 90% of advanced colorectal cancers and contributes to the malignant characteristics of tumors (18). However, it is not useful in detecting asymptomatic cancer, as the sensitivity of CEA determination for early colorectal cancer is as low as 30–40% (19). Moreover, CEA is not significantly associated with survival in patients with stage I and II lesions, and CEA testing is relatively insensitive to tumors with local or peritoneal involvement (20). Therefore, the identification of sensitive prognostic markers in this subgroup would allow for the use of postoperative adjuvant therapy in a subset of patients with poor prognosis and ultimately improve survival.

In this study, we aimed to determine whether CRP provides more accurate prognostic information than that offered by the existing staging systems or tumor markers in stages I–III, or in subgroups of stages II or III colorectal cancer patients. We revealed that CRP was significantly associated with serosal invasion, preoperative CEA levels and TNM classification, which are established conventional prognostic factors. Furthermore, CRP positivity was found to have independent prognostic value in stages I–III, whereas the prognostic values of CEA or TNM classification were affected by other clinical factors.

Studies on various types of malignancies have emphasized the importance of examining multiple lymph nodes in determining prognosis. In colon and rectal cancer, staging accuracy and survival are improved by increasing the number of nodes examined and analyzed (2,21,22). In addition, failure to examine a sufficient number of lymph nodes may result in the inability to identify patients in whom lymph nodes are affected by cancer, thus resulting in understaging (23). However, the number of lymph nodes reported with colectomy varies widely and may be due to variations in surgical technique, the thoroughness of the pathologist in finding nodes in the specimen, or the actual number of regional lymph nodes. Therefore, it is crucial to establish the minimum number of lymph nodes required for an acceptable accuracy in classifying a tumor as LN-negative. Current guidelines established by the AJCC recommend the assessment of 12 or more nodes for accurate staging (8).

Nonetheless, the agenda for adequate lymph node evaluation remains debatable. Recently, published studies assessing the number of lymph nodes resected in colorectal cancer have reported wide variation in the extent of resection. Although these studies demonstrate a prognostic association between the number of lymph nodes examined and survival, the cut-off values vary widely, ranging from 6 to 40 (11). These differences can be attributed to the fact that the right side of the colon is associated with a higher number of lymph nodes retrieved compared with the left (3,9,24). In addition, older age and obesity may reduce the number of lymph nodes retrieved (3,9). The number of lymph nodes that can be retrieved may depend on the immune response of the patient as the size and morphology of lymph nodes are modified by immune response (25,26).

Our results revealed that the optimal cut-off value for the lymph node number retrieval is 12 in stage II, but no statistical difference was observed between the number of patients in the ≤12 and the >13 node groups. However, in patients with ≤12 nodes retrieved, CRP-positive patients had a significantly poorer prognosis than CRP-negative patients. By contrast, the cancer-specific survival rate of CRP-positive patients was the same as that of CRP-negative patients in those with >13 nodes counted. Therefore, CRP evaluation may be a useful approach for the detection of patients with the possibility of stage migration caused by sub-optimal lymph node examination.

The most widely used staging system is the TNM staging system, which was proposed by the AJCC/UICC. In the sixth edition, pN stage was stratified into pN1 (LN 1–3) and pN2 (LN ≥4) according to the number of positive lymph nodes (27). However, this system is also affected by the total number of lymph nodes harvested and examined (2,4,28). To overcome these variables, a ratio-based node staging system has been proposed. The LNR is defined as the number of positive lymph nodes divided by the total number of lymph nodes examined. It was demonstrated that the ratio-based classification was superior to the traditional categorical pN stage in gastric, pancreatic and breast cancer (29–31). LNR reflects the probability of positive lymph nodes in the harvested nodes, which does not significantly depend on the number of lymph nodes harvested.

The LNR has been demonstrated to have prognostic value in colon cancer in various studies (33–35). Data for rectal cancer were limited but Rosenberg *et al* reported that, following subgroup analysis, the LNR was an independent prognostic factor for cause-specific survival in patients with rectal cancer (36). In addition, Peng *et al* found that LNR was the most significant factor for overall survival, disease-free survival and local recurrence in patients with rectal cancer (37). However, there is no consensus concerning LNR, as different techniques for its calculation have been described. With more studies demonstrating the importance of LNR, the pooling of data may help to determine the proper methodology for LNR stratification.

In our study of patients with stage III colorectal cancer, the LNR (>0.15) and preoperative CRP status were independent prognostic factors for cancer-specific survival, while the conventional categorical pN stage (LN ≥4) and the total harvested lymph node number (>13) were not significant in the multivariate analysis. This confirmed the stronger prognostic value of the LNR or preoperative CRP status in patients with stage III colorectal cancer. Furthermore, combining the LNR or harvested lymph node number and the preoperative CRP status may provide more accurate data for predicting poor prognosis and identify patients requiring further, intense chemotherapy in stage III colorectal cancer. In the patients with LNR ≤0.15 or ≤12 lymph nodes retrieved, CRP-positive patients had a significantly poorer prognosis than CRP-negative patients. By contrast, cancer-specific survival in CRP-positive patients was the same as that of CRP-negative patients in those with LNR >0.15 or >13 nodes retrieved.

In conclusion, preoperative CRP provides an independent prognostic value for colorectal cancer in stages I–III and is superior to the currently used number of lymph nodes harvested or pN stage. Although the sample size included in this study is small when compared with other multicenter or population-based studies, its prognostic power does not depend on the number of lymph nodes harvested. With the combination of CRP status and total harvested number of lymph nodes in stages II or III colorectal cancer, better stratification may aid in identifying high-risk patients in order that adjuvant therapy be tailored to increase the number of positive patient outcomes.

## Figures and Tables

**Table I:** Correlation between CRP status and clinicopatho logical characteristics of stage I–III patients undergoing potentially curative resection for colorectal cancer.

Factors	CRP >0.5	CRP <0.5	p-value
Age			
>66	32	64	
<66	13	84	0.0019
Gender			
Male	19	90	
Female	26	58	0.8493
T-stage			
I–II	8	50	
III–IV	37	98	0.0622
T4 tumor			
Negative	38	141	
Positive	7	7	0.0337
Venous invasion			
Negative	20	76	
Positive	25	72	0.5214
Lymphatic invasion			
Negative	3	16	
Positive	42	132	0.5951
Lymph node metastasis			
Negative	28	89	
Positive	17	59	0.9388
Pathology			
Differentiated	43	135	
Undifferentiated	2	13	0.526
TNM classification			
I	6	36	
II	22	52	
III	17	60	0.1585
CEA			
<0.5	50	98	
>0.5	29	16	0.0005

CRP, C-reactive protein; CEA, carcinoembryogenic antigen.

**Table II:** Uni- and multivariate Cox’s proportional hazard model for cancer-specific survival in stage I–III colorectal cancer.

Factors	HR	95% CI	p-value
Univariate analysis			
CRP (>0.5)	5.03	2.49–10.18	<0.0001
CEA (>5)	3.43	1.62–7.25	0.0008
Total number of dissected lymph nodes (<12)	1.92	0.89–4.42	0.08
Lymph node metastasis (positive)	1.72	0.85–3.46	0.13
T-stage (III, IV)	3.17	1.11–9.01	0.03
Lymphatic invasion (positive)	3.46	0.48–25.11	0.22
Vessel invasion (positive)	1.31	0.65–2.65	0.45
Pathology (undifferentiated type)	3.08	1.27–7.51	0.013
Multivariate analysis			
CRP (>0.5)	4.90	2.33–10.32	<0.0001
CEA (>5)	2.15	0.98–4.72	0.054
T-stage (III, IV)	2.02	0.62–5.96	0.21
Pathology (undifferentiated type)	4.25	1.68–10.76	0.002

CRP, C-reactive protein; CEA, carcinoembryogenic antigen. HR, hazard ratio; CI, confidence interval.

**Table III:** Uni- and multivariate Cox’s proportional hazard model for cancer-specific survival in stage II colorectal cancer.

	Univariate analysis			Multivariate analysis		
	
Factors	HR	95% CI	p-value	HR	95% CI	p-value
CRP (>0.5)	5.20	1.64–16.48	0.005	4.20	1.26–14.00	0.02
CEA (>5)	1.61	0.52–5.06	0.41	-	-	-
Total number of dissected lymph nodes (>12)	0.29	0.06–1.33	0.11	-	-	-
T4 tumor	4.00	1.21–13.30	0.024	2.55	0.73–8.93	0.15
Lymphatic invasion (positive)	ns	ns	0.40	-	-	-
Vessel invasion (positive)	2.25	0.61–8.29	0.23	-	-	-
Pathology (undifferentiated type)	2.86	0.60–12.97	0.18	-	-	-

CRP, C-reactive protein; CEA, carcinoembryogenic antigen; ns, not significant; HR, hazard ratio; CI, confidence interval.

**Table IV:** Uni- and multivariate Cox’s proportional hazard model for cancer-specific survival in stage III colorectal cancer.

	Univariate analysis			Multivariate analysis		
	
Factors	HR	95% CI	p-value	HR	95% CI	p-value
CRP (>0.5)	3.80	1.41–10.21	0.008	3.11	1.13–8.55	0.028
CEA (>5)	4.76	1.36–16.63	0.015	3.49	0.97–12.55	0.057
Total number of lymph nodes examined (>12)	2.28	0.83–6.27	0.11	-	-	-
LNR (>0.15)	2.32	1.07–5.02	0.033	2.26	0.78–6.54	0.2262
pN2 vs. pN1	0.73	0.21–2.56	0.63	-	-	-
Pathology (undifferentiated type)	2.46	0.70–8.64	0.16	-	-	-
T4 tumor (positive)	2.48	0.91–6.80	0.079	-	-	-

CRP, C-reactive protein; CEA, carcinoembryogenic antigen. HR, hazard ratio; CI, confidence interval.
